# Distinct clinical and prognostic implication of IDH1/2 mutation and other most frequent mutations in large duct and small duct subtypes of intrahepatic cholangiocarcinoma

**DOI:** 10.1186/s12885-020-06804-6

**Published:** 2020-04-15

**Authors:** Bingqi Ma, Huijuan Meng, Ye Tian, Yingying Wang, Tianqiang Song, Ti Zhang, Qiang Wu, Yunlong Cui, Huikai Li, Wei Zhang, Qiang Li

**Affiliations:** 1 Department of Hepatobiliary Surgery, Tianjin Medical University Cancer Institute and Hospita; National Clinical Research Center for Cancer; Key Laboratory of Cancer Prevention and Therapy, Tianjin; Tianjin’s Clinical Research Center for Cancer, Tianjin, 300060 China; 2 Affiliated Hospital of Weifang Medical University, Weifang, 261031 China; 3 Tianjin Medical University Cancer Institute and Hospital; National Clinical Research Center for Cancer; Key Laboratory of Cancer Prevention and Therapy, Tianjin; Tianjin’s Clinical Research Center for Cancer, Tianjin, 300060 China

**Keywords:** Intrahepatic cholangiocarcinoma (ICC), IDH1/2 mutation, BAP1, ARID1A, PBRM1, Large duct type, Small duct type

## Abstract

**Background:**

Isocitrate dehydrogenase 1/2 (IDH1/2), BAP1, ARID1A and PBRM1 have been reported as the most frequent mutant genes in intrahepatic cholangiocarcinoma (ICC), and their relationships with clinicopathological features and prognosis were researched in this study.

**Methods:**

We collected clinical data of 130 ICC patients from January 2012 to December 2017. The IDH1/2 mutation and loss of BAP1, ARID1A and PBRM1 expressions were detected by DNA sequencing or immunohistochemical methods, and histological subtype of ICCs was determined by hematoxylin-eosin, Alcian blue and S100P staining.

**Results:**

IDH1/2 mutation was related to decreased preoperative serum total bilirubin (*P* = 0.039), ferritin (*P* = 0.000) and higher histological differentiation (*P* = 0.024), and was associated with prolonged disease-free survival (*P* = 0.009) and a trend toward increased overall survival (*P* = 0.126) in small duct type of ICCs. Immunohistochemical staining results of MsMab-1 were generally consistent with DNA sequencing for IDH1/2 mutant in ICCs (κ = 0.691). Only BAP1 expression loss was correlated to prolonged disease-free survival (*P* = 0.031) and overall survival (*P* = 0.041) in large duct type of ICCs.

**Conclusions:**

IDH1/2 mutation is a favorable predictor and may be related to iron metabolism in small duct type of ICCs. Furthermore, we suggest that the detection of IDH1/2 mutation is indispensable to determine targeted therapy in small duct type ICCs, while it is not necessary in large duct of ICCs. MsMab-1 is a relatively effective multi-specific antibody against IDH1/2 mutant in ICCs. BAP1 expression loss was correlated with improved prognosis only in large duct type ICCs.

## Background

Tumors originated from the bile ducts can be subdivided anatomically into three subgroups: distal, perihilar, and intrahepatic cholangiocarcinoma (ICC). The ICCs represent approximately 20% of bile duct malignancies and account for 15–20% of primary liver cancers [1]. ICC is related to a dismal prognosis with a postoperative 5-year overall survival (OS) rates of 17% [2]. Meanwhile, the incidence of ICC is increasing in recent years [3]. With the application of next-generation sequencing technology, many frequent genetic mutations have been discovered in ICCs, such as isocitrate dehydrogenase 1/2 (IDH1/2), KRAS, BAP1, ARID1A, PBRM1 and FGFR2-fusion [4, 5]. Almost all literatures reported that the incidences of their mutations are more than 10% in cases with ICCs [4–9].

In fact, the distinction between ICC and perihilar cholangiocarcinoma at second-order biliary branches is someway artificial and seems to take in consideration of surgical implications rather than molecular, histological and anatomical aspects [10]. As proposed by several investigators [10–15], ICCs are much heterogeneous and can be histologically classified into two subtypes according to the size of displayed bile duct: large duct type and small duct type (also known as bile duct type and cholangiolar type). Moreover, this category individuates ICC subgroups with different clinical and molecular features [12, 13]. For example, large duct type ICC representatively lacks IDH1/2 mutation and FGFR2-fusion, characteristics exclusively seen in small duct ICC [14]. Whereas, KRAS mutation was detected in 23% of large duct type and 1% of small duct type ICC respectively (*P* = 0.00003) [15].

IDH1 and IDH2 genes encode the NADP^+^-dependent isocitrate dehydrogenase in the cytoplasm and mitochondria respectively, catalyzing the conversion from isocitrate to α-ketoglutarate (α-KG). IDH1/2 mutations have been detected in many human cancers, including glioma, chondrosarcoma, acute myeloid leukemia, and cholangiocarcinoma [16–19]. These mutations acquire neomorphic activity that transforms α-KG into oncometabolite 2-hydroxyglutarate (2-HG). 2-HG accumulation may inhibit specific α-KG dependent dioxygenases and participate in tumorigenesis involving cell signaling, extracellular matrix maturation, and epigenetic regulation [20, 21]. Although previous studies have researched the association of IDH mutation with prognosis and pathology in ICC patients, the data are conflicting [4, 6, 7].

BAP1, ARID1A and PBRM1 represent the very frequently mutated histone modifying and chromatin remodeling genes in human cancer, such as renal cell carcinoma, ovarian carcinoma and ICC [4, 22, 23]. Due to the diversity of mutational hot-spots and the large scales of these genes, detection of these mutations is relatively difficult compared with IDH or KRAS. Alternatively, immunohistochemistry (IHC) staining is effective to detect the above mutations, because the presence of mutations correlates strongly with loss of the corresponding protein expressions [22, 24]. Some studies have estimated the association of these inactive mutations with clinical features and prognosis, but there are still many discrepancies [4, 8, 9, 25].

Here we investigated the mutational status of IDH1/2, BAP1, ARID1A and PBRM1, and for the first time, determined whether these mutations are correlated to clinicopathological characteristics and prognoses in each histological subtype of ICCs. Due to the heterogeneity of ICC, by analyzing their relationships in the subtypes, rather than the total cohort of ICCs, we could obtain more reasonable and accurate results to explain the inconsistencies reported previously [4, 6–9, 25].

## Methods

### Patients and tissue samples

For this study, we selected 130 consecutive patients with ICC who underwent surgical treatment at Tianjin Medical University Cancer Institute and Hospital from January 2012 to December 2017. Because of the challenge of determining exact anatomical origin of some tumors, the ICCs with similar features of perihilar carcinoma were excluded. Cases with combined HCC-CCA, composed of typical HCC and typical ICC, were excluded. All patients were reviewed to confirm the diagnosis of ICC and to restage according to the 8th edition 2017 AJCC staging system. After exclusion of 28 patients for prognostic analysis (including 11 cases lost to follow-up, 14 cases received non-R0 resection, 1 cases died of postoperative complications, and 2 cases died of some non-tumor-related causes), 130 and 102 patients were eventually used for comparisons of clinical characteristics and survival analyses respectively.

Formalin-fixed paraffin-embedded (FFPE) samples and hematoxylin-eosin (HE) staining slides of 130 surgical specimens were collected from Pathology Department of Tianjin Medical University Cancer Institute and Hospital. Tissue microarrays (TMAs) composed of 2-mm cores of FFPE tumor tissue were constructed for various staining by selecting a representative tumor areas and a typical peritumoral region from each case.

### Clinical data

Clinical data, including the patients’ gender, age, surgical records, imaging examinations and laboratory tests were carefully reviewed and analyzed. All cases were regularly followed up every 3 months for tumor recurrence by screening of plasma tumor markers and imaging examinations. Disease-free survival (DFS) was measured from the date of surgery to the date of first recurrence or last follow-up, whereas OS was defined as the interval between the date of first diagnosis of ICC and the date of death or last follow-up.

### Histopathology

HE staining was performed routinely on a set of TMAs. According to histological features, we subclassified ICCs into two types: large duct and small duct [11, 14, 15].

**Fig. 1:**
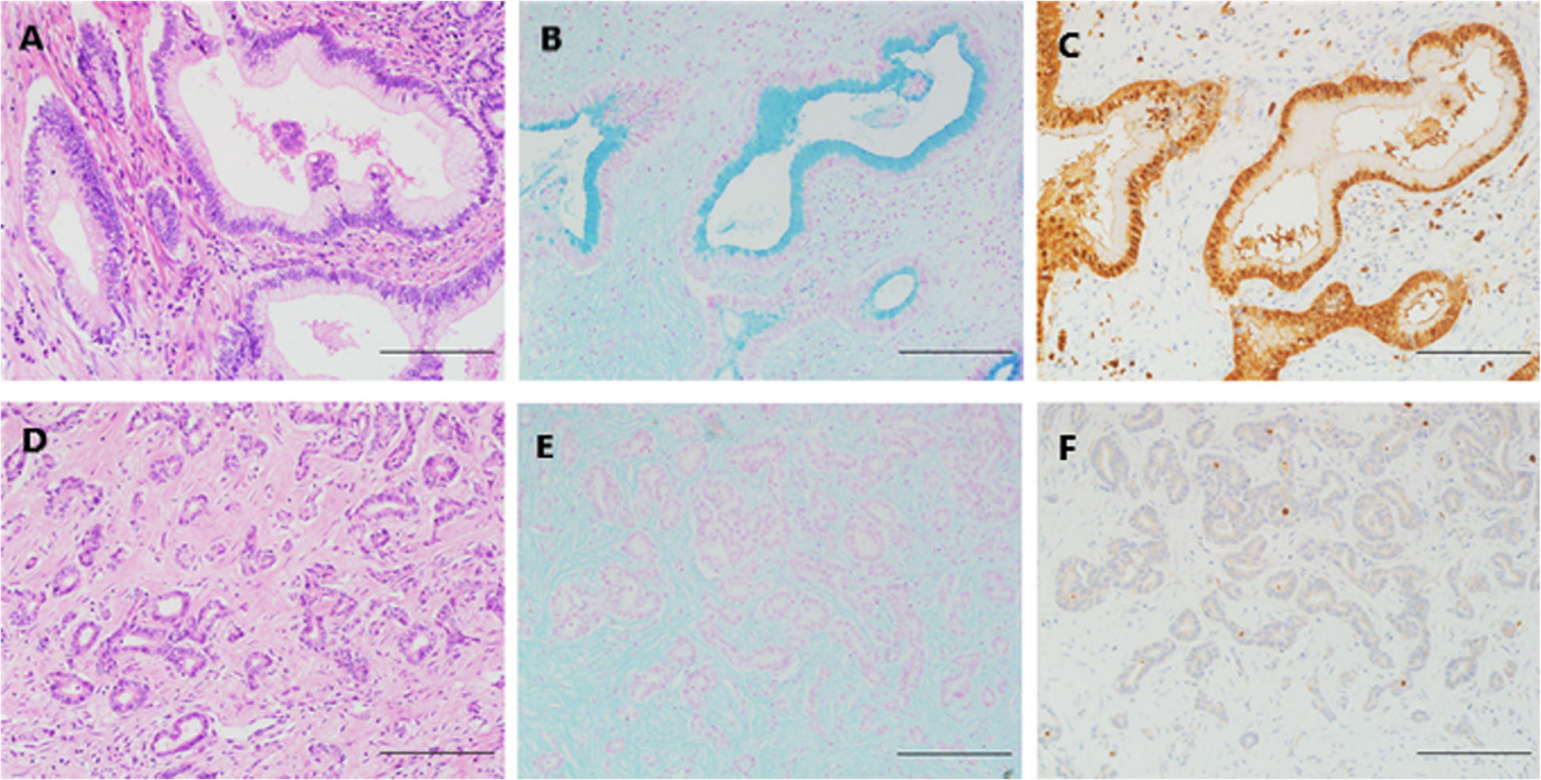
The representative histology, mucin production and S100P expression of large duct type (**a**-**c**) and small duct type (**d**-**f**) of ICCs. These images were stained by HE (**a**, **d**), Alcian blue (**b**, **e**), and S100P (**c**, **f**), respectively. Scale bar represents 200 um

Type1 (large duct type) is made up of tall columnar tumor cells arranged in a large-sized glandular or tubular pattern and usually shows abundant desmoplastic stroma and low cellularity. Meanwhile, the tumor cells of type1 contain abundant eosinophilic, clear, or mucinous cytoplasm, and their nuclei are usually high grade. Extracellular mucin can be detected in glandular spaces (Fig. 1a).

Type2 (small duct type) has histological features of small-sized tubular or acinar component composed of low columnar to cuboidal tumor cells and usually shows scant stroma and high cellularity. This type can also be arranged in cribrate or solid patterns. The tumor cells of Type2 have a higher nuclear-to-cytoplasmic ratio compared with type1 tumors and usually contain scanty amphophilic or eosinophilic cytoplasm (Fig. 1d).

If there was any doubt in the process of classification, we can refer to whole mount sections for further observation. Ultimately, some cases, not clearly classified, were termed undetermined type.

Mucin production was stained by Alcian Blue Stain Kit (pH 2.5) (G1560; Solarbio Science & Technology Co., Ltd., Beijing, China) on TMAs to distinguish subtypes of ICCs (Fig. 1b,e). Alcian blue staining was scored semiquantitatively by the proportion of glandular lumens with mucin production on a scale from 0 to 2: score 0, < 10%; score 1, 10 to 50%; and score 2, > 50% or frequent intracytoplasmic mucin [10, 26].

### Immunohistochemistry

TMAs sections were dewaxed and rehydrated in xylene and gradient ethanol, respectively. After antigen retrieval and endogenous peroxidase activity blocking, the slides were incubated with primary antibodies (4 °C for 14 h and 37 °C for 1 h) and HRP-conjugated secondary antibody (37 °C for 1 h) in turn. Then the sections were visualized with 3,3′-diaminobenzidine (ZLI-9017; Zhongshan Goldbridge) for 5 min and counterstained with Hematoxylin. Appropriate internal or external positive controls and negative controls were designed and used for each round.

The primary antibodies used in the present study were as follows: S100P (clone EPR6142; dilution 1:300; Abcam, Cambridge, UK), IDH1/2 mutant (R132/172) (clone MsMab-1; dilution 1:200; EMD Millipore Corp., Billerica, MA, USA), BAP1 (ab199396; dilution 1:200; Abcam), ARID1A (clone EPR13501–73; dilution 1:500; Abcam), PBRM1 (HPA015629; dilution 1:200; Sigma-Aldrich).

The expression of S100P showed nuclear and cytoplasmic staining pattern and was evaluated semiquantitatively according to the percentage of positive tumor cells as follows: score 0, < 1%; score 1, 1 to 25%; score 2, 26 to 50%; score 3, 51 to 75%; and score 4, 76 to 100% [10]. (Fig. 1c,f).

**Fig. 2:**
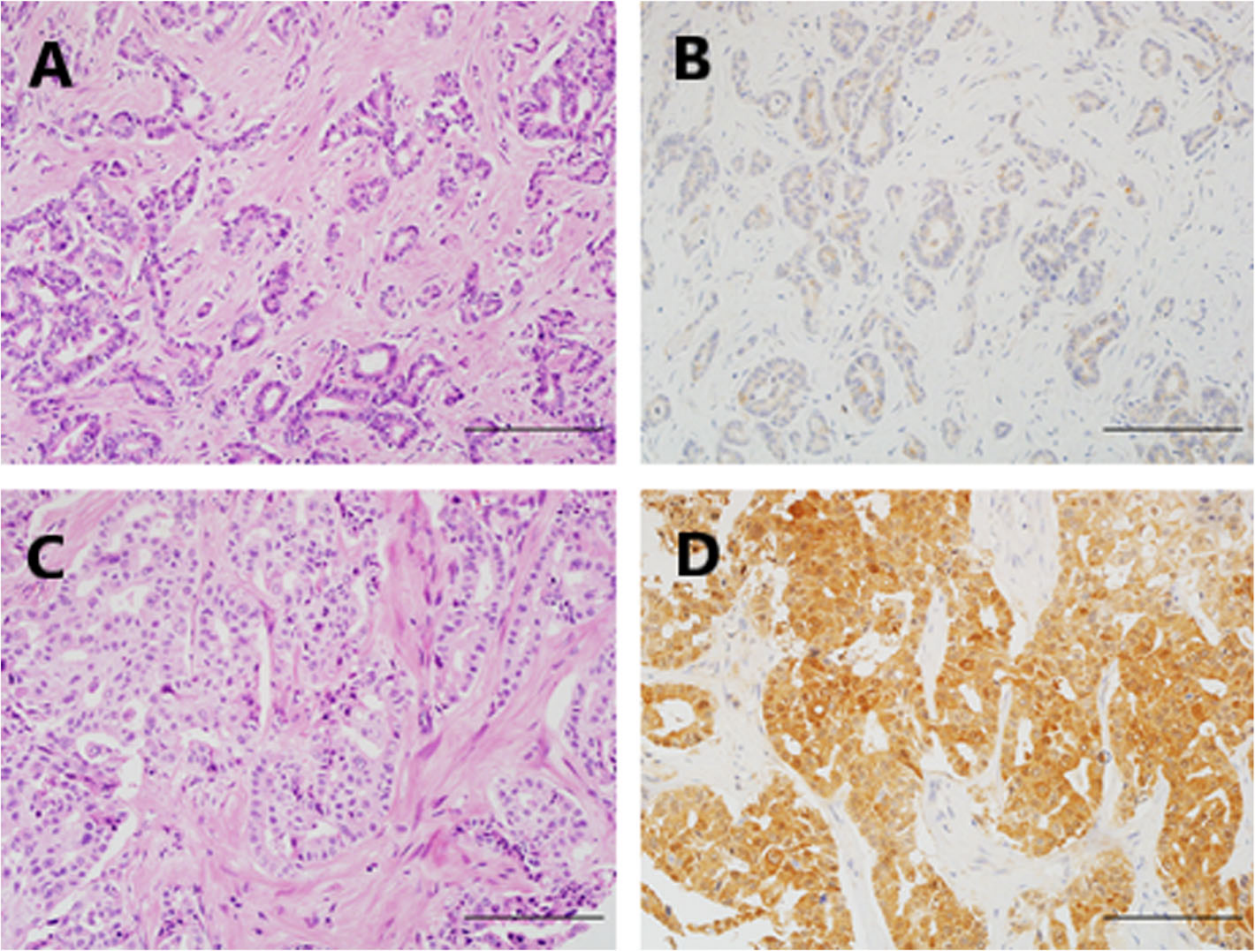
The representative HE and IHC images of IDH1/2 mutant negative (**a**, **b**) and positive (**c**, **d**) staining in cases with ICC. These corresponding images of HE (**a**, **c**) were presented as control of each IHC staining (**b**, **d**), respectively. Scale bar represents 200 um

**Fig. 3:**
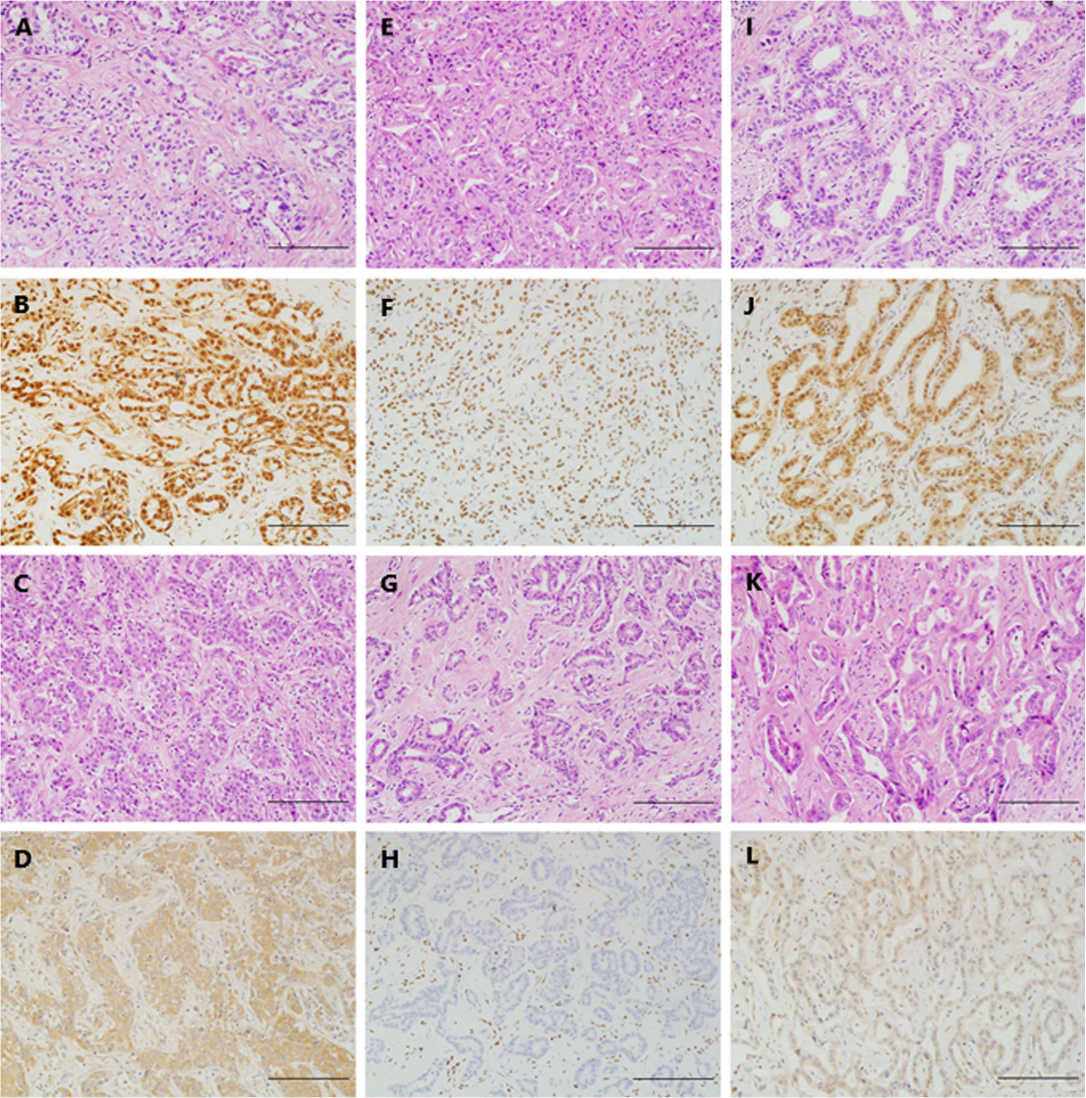
The representative HE and IHC images of BAP1 (**a**-**d**), ARID1A (**e**-**h**) and PBRM1 (**i**-**l**) expression in cases with ICC. The nuclear staining was detected in cases with the normal expression of BAP1 (**b**), ARID1A (**f**) and PBRM1 (**j**). Similarly, the loss of BAP1 (**d**), ARID1A (**h**) and PBRM1 (**l**) expression were interpreted as inactive mutations of these genes. These corresponding images of HE (**a**, **c**, **e**, **g**, **i**, **k**) were presented as control of each IHC staining (**b**, **d**, **f**, **h**, **j**, **l**), respectively. Scale bar represents 200 um

IDH1/2 mutant exhibited cytoplasmic and mild nuclear expression, while only nuclear staining was interpreted as immunopositive in the evaluation of BAP1, ARID1A, and PBRM1 expression (Figs. 2 and 3). A semiquantitative scoring system was used for the above 4 factors in terms of the proportion of positive neoplastic cells: score 0 (negative), < 1%; score 1 (focal or regional positive), 1 to 90%; score 2 (diffuse positive), 91 to 100% [27, 28].

### Analysis of IDH1/2 mutation

Genomic DNA of ICCs was extracted from ten 10-um-thick sections micro-dissected from FFPE tissue blocks. Two pairs of primers were designed for hotspot mutations of IDH1/2 [10]. The first pair of primers for IDH1-R132: forward 5′- ACACGACGCTCTTCCGATCTACACATACAAGTTGGAAATTTCTGG-3′ and reverse 5′- GACGTGTGCTCTTCCGATCTAATCACCAAATGGCACCATAC-3′; the second pair of primers for IDH2-R140 and IDH2-R172: forward 5′-ACACGACGCTCTTCCGATCTCAGAGACAAGAGGATGGCTAGG-3′ and reverse 5′-GACGTGTGCTCTTCCGATCTTGTCCTCACAGAGTTCAAGCTG-3′ (Tsingke Biological Technology CO., Ltd., Beijing, China). The target DNA sequences were amplified by polymerase chain reaction using the primers above, and were further sequenced and analyzed for IDH1/2 mutation.

### Statistical analysis

Categorical variables were presented as totals and frequencies, and evaluated by chi-square test or Fisher exact test, as appropriate; Continuous variables were described as means with standard deviations or medians with ranges, and compared with t test or Mann-Whitney U test, as appropriate. DFS and OS were calculated by Kaplan-Meier method, and assessed by log-rank test for univariate analysis. Cox proportional hazards model was conducted to adjust for the bias from tumor characteristics (e.g. TNM stage, bilateral involvement) in analyzing prognostic value of gene mutations and other clinical factors. The results were recorded as hazard ratios (HR) and 95% confidence intervals (CIs). A two-tailed *P* value less than 0.05 was considered significant. SPSS statistical program (IBM SPSS Statistics 24, Chicago, IL, USA) was used for data analysis.

## Results

### Large duct and small duct type of ICCs

#### Subclassification of ICCs

We classified 130 cases with ICC into 3 subtypes on the basis of histological features, S100P expression and Alcian blue staining in the first round. 12 cases (9.2%) were recognize as typical large duct type, which met three standards: HE, type1; S100P, score 3–4; and Alcian blue, score 1–2. Meanwhile, 73 cases (56.2%) were typical small duct type, which satisfied the following conditions: HE, type2; S100P, score 0–1; and Alcian blue, score 0–1. The remaining 45 cases (34.6%) were intermediate type that exhibited mixtures of two subtypes or indeterminate features (Supplementary Table S1).

In the second round, according to the above criteria, we set classification efficiency of three factors as follows: HE > S100P > Alcian blue. Then 45 cases of intermediate type were divided into 2 subcategories (Supplementary Table S2). Finally, we identified 27 (20.8%) and 103 (79.2%) cases as large duct and small duct type ICCs, respectively (Table 1) The concordant rate and κ value of interpretations between two observer were 96.9% and 0.904, respectively.

**Table 1:** Integrated results of the ICC subclassification

**Subtype**	**HE**			**AB**			**S100P**				
	Large	Small	underdetermined	0	1	2	0	1	2	3	4
**Large duct**	12	0	15	3	6	18	0	2	0	10	15
**Small duct**	0	86	17	92	8	3	68	20	6	9	0

#### Clinical characteristics and prognosis of ICC subtypes

Our study cohort included 71 males (54.6%) and 59 females (45.4%), with a mean age of 57.8 ± 9.7 years (range from 28 to 77). 71 cases (54.6%) underwent lymphadenectomy.

The large duct type was more likely to have higher level of CA19–9 (*P* = 0.002), higher frequencies of lymphadenectomy (P = 0.002), nerve invasion (*P* = 0.025), satellite lesions (*P* = 0.009), smaller size of tumor (*P* = 0.021) and more aggressive tumor stage of pT (*P* = 0.041), pM (*P* = 0.019), and TNM classification (P = 0.04) than small duct type (Supplementary Table S3).

The median follow-up period of 102 ICC cases was 25.2 months (range from 4.9 months to 100.0 months). Although patients with large duct type had worse prognosis than those with small duct type in univariate analysis (DFS, *P* = 0.063; OS, *P* = 0.031) (Figure S1), the histological subtype was not an independent prognostic factors of ICCs in multivariate analysis (*P* > 0.10).

### IDH1/2 mutations in ICCs

#### IDH1/2 mutations by DNA sequencing and IHC

DNA sequencing showed 21 patients (16.1%) harbored IDH1/2 mutation, including IDH1-R132C, IDH1-R132G, IDH1-R132H, IDH1-R132L, IDH2-R172K, and IDH2-R172W. But no mutation of IDH2-R140 was detected. Meanwhile, IDH1/2 mutant was detected in 14 cases (10.8%) by MsMab-1, which is specific for IDH1-R132G, IDH1-R132H, and IDH2-R172W according to instructions (Table 2). IHC analysis verified the specificity of MsMab-1 according to results detected by DNA sequencing, and showed that sensitivity and specificity of MsMab-1 to detect specific types of IDH1/2 mutation were 81.8% (9 of 11) and 95.8% (114 of 119), respectively. Accordingly, MsMab-1 was a relatively effective multi-specific antibody against IDH1/2 mutant in ICCs (κ = 0.691).

**Table 2:** Comparisons of IDH1/2 mutations in ICCs between DNA sequencing and IHC methods

**DNA sequencing**				**MsMab-1 staining**		
**Mutant gene**	Nucleotide change	Amino acid change	Number	Positve	Negative	Reactivity by manual
**IDH1**	CGT > TGT	R132C	6	1	5	No
**IDH1**	CGT > GGT	R132G	5	4	1	Yes
**IDH1**	CGT > CAT	R132H	4	4	0	Yes
**IDH1**	CGT > CTT	R132L	2	1	1	No
**IDH2**	AGG > TGG	R172W	2	1	1	Yes
**IDH2**	AGG > AAG	R172K	2	0	2	No
**Wild type**			109	3	106	No

#### Clinical implications of IDH1/2 mutation in small duct type of ICCs

IDH1/2 mutation was detected in 3.7% (1/27) of cases with large duct type and 19.4% (20/103) of patients with small duct type, respectively. Patients with IDH1/2 mutation had decreased TBIL (total bilirubin) (*P* = 0.039), Fe (ferritin) (*P* = 0.000) and higher histological differentiation (*P* = 0.024) in small duct type (Table 3).

**Table 3:** Comparisons of clinicopathological characteristics between IDH1/2 mutant and wild type of ICCs

**Clinical variables**	**Total cohort**			**Small duct type**		
	IDH1/2 mutant type	IDH1/2 wild type	***P*-valve**	IDH1/2 mutant type	IDH1/2 wild type	***P*-valve**
**TBIL** (umol/L)			0.122			**0.039**
< 21	20 (95.2%)	85 (78.7%)		20 (100.0%)	65 (79.3%)	
≥ 21	1 (4.8%)	23 (21.3%)		0 (0.0%)	17 (20.7%)	
**INR**			**0.025**			0.054
<1.0	17 (81.0%)	59 (54.6%)		16 (80.0%)	47 (56.6%)	
≥ 1.0	4 (19.0%)	49 (45.4%)		4 (20%)	36 (43.4%)	
**CEA** (ug/L)			0.162			0.067
< 5	19 (90.5%)	82 (75.9%)		19 (100.0%)	68 (81.9%)	
≥ 5	2 (9.5%)	26 (24.1%)		0 (0.0%)	15 (18.1%)	
**Fe** (ug/L)			**0.000**			**0.000**
< 200	17 (85.0%)	42 (41.2%)		17 (89.5%)	34 (44.2%)	
≥ 200	3 (15.0%)	60 (58.8%)		2 (10.5%)	43 (55.8%)	
**Histological differentiation**			**0.028**			**0.024**
Low	5 (23.8%)	53 (50.0%)		5 (25.0%)	44 (53.0%)	
Moderately to high	16 (76.2%)	53 (50.0%)		15 (75.0%)	39 (47.0%)	
**Histological subtype**			0.074			
Large duct	1 (4.8%)	26 (23.9%)				
Small duct	20 (95.2%)	83 (76.1%)				

**Fig. 4:**
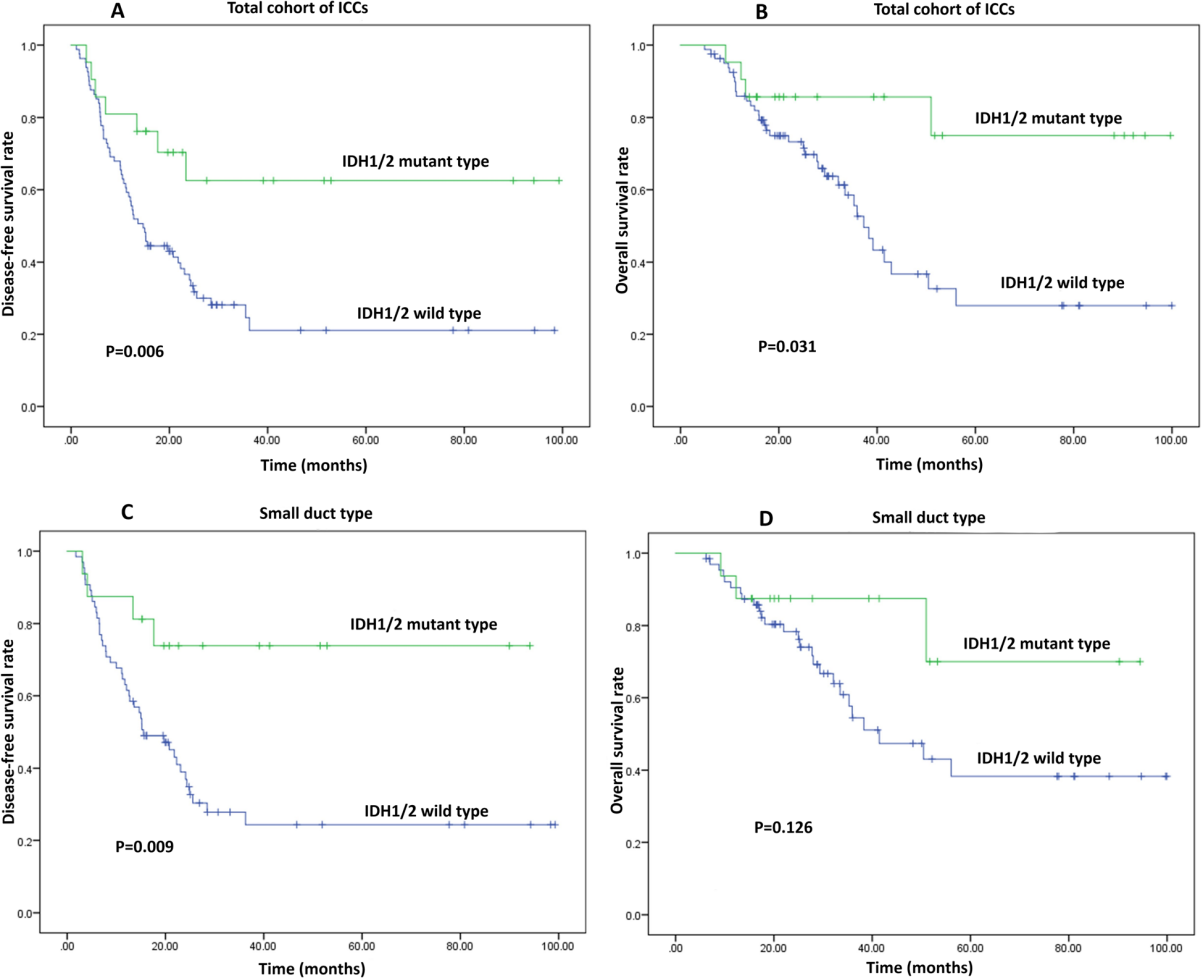
Kaplan-Meier curves showing patients with IDH1/2 mutation had prolonged DFS (**a**) and OS (**b**) in total cohort of ICCs; and significant prolonged DFS (**c**) and a trend toward increased OS (**d**) were associated with IDH1/2 mutation in small duct type of ICCs

Univariate and multivariate analyses exhibited that cases with IDH1/2 mutation had significantly improved prognosis than those without IDH1/2mutation in terms of DFS (*P* = 0.006) and OS (*P* = 0.031) in total cohort of ICCs (Fig. 4a-b, Supplementary Table S4). And more notably, Kaplan-Meier analysis showed IDH1/2 mutation was associated with prolonged DFS (*P* = 0.009) and a trend toward increased OS (*P* = 0.126) in small duct type of ICCs. Further multivariate analysis confirmed IDH1/2 mutation was a favorable independent predictor of DFS (*P* = 0.022, HR = 3.452, 95% CI = 1.191–10.004), and was not significantly related to OS (*P* = 0.114, HR = 2.686, 95%CI = 0.789–9.144) in patients with small duct type (Fig. 4c-d, Table 4).

**Table 4:** Univariate and multivariate analyses for prognostic value of frequent mutations in the subtypes of ICCs

**Clinical variables**	**DFS in large duct type**		**OS in large duct type**		**DFS in small duct type**		**OS in small duct type**	
	Univariate *P*-value	Multivariate *P*-valve/ HR (95% CI)	Univariate *P*-value	Multivariate *P*-valve/ HR (95% CI)	Univariate *P*-value	Multivariate *P*-valve/ HR (95% CI)	Univariate *P*-value	Multivariate *P*-valve/ HR (95% CI)
**IDH1/2**					**0.009**	**0.022**	0.126	0.114
Mutant vs. wild						3..452 (1.191–10.004)		2.686 (0.789–9.144)
**BAP1**	**0.031**	0.059	**0.041**	**0.039**	0.952		0.454	
Loss vs. retained		1.711 (0.979–2.990)		2.985 (1.038–4.186)				
**ARID1A**	0.432		0.580		0.357		0.626	
Loss vs. retained								
**PBRM1**	0.672		0.159		0.284		0.614	
Loss vs. retained								

### Expression status of BAP1, ARID1A and PBRM1 in ICCs

#### Loss of BAP1, ARID1A and PBRM1 expressions

BAP1 expression was retained in 72 cases (55.4%), while 47 cases (36.2%) and 11 cases (8.5%) showed complete and focal loss of BAP1 expression respectively. Complete or regional loss of ARID1A and PBRM1 staining was observed in 28 cases (21.5%) and 45 cases (34.6%), respectively. (Supplementary Table S5) There was obviously positive correlation among their expression loss (*P* < 0.05), especially between BAP1 and PBRM1 (*P* = 0.000) (Supplementary Table S6).

#### Clinical implications of BAP1, ARID1A and PBRM1 expression loss in subtypes of ICCs

The loss of BAP1 expression was associated with decreased ALT (alanine aminotransferase), AST (aspartate aminotransferase), TBIL and higher histological differentiation in large type of ICCs, while it was correlated to larger size of tumor in small duct type (Supplementary Table S7). All clinical factors significantly related to the loss of ARID1A expression, were within small duct type of ICCs, such as decreased ALT, AST, lower incidence of liver cirrhosis and earlier TNM stage (Supplementary Table S8). PBRM1 loss correlated decreased AST, TBIL, IBIL (indirect bilirubin), and CA19–9 in the large duct type of ICCs (Supplementary Table S9). In addition, the loss of BAP1, ARID1A and PBRM1 expressions had similar distribution in large duct and small duct type of ICC patients (*P* > 0.05) (Supplementary Table S7–9).

**Fig. 5:**
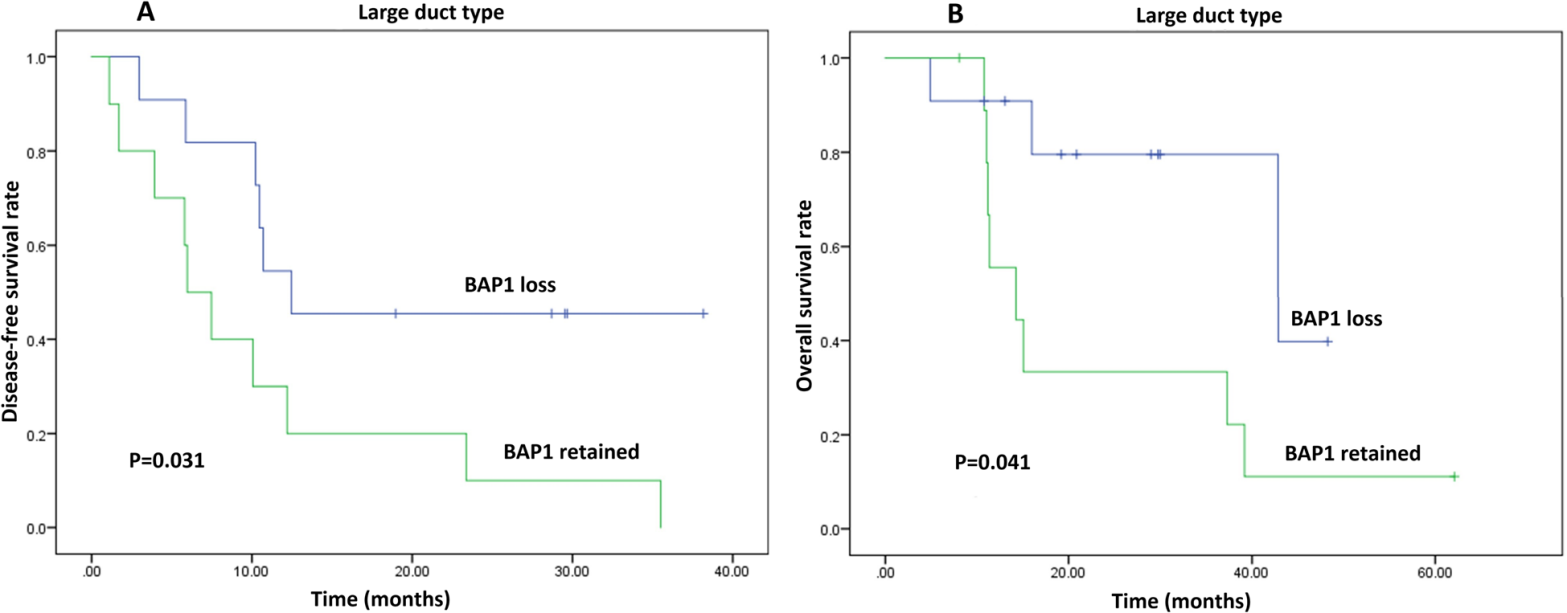
Kaplan-Meier curves showing patients with loss of BAP1 had improved DFS (**a**) and OS (**b**) in large duct type of ICCs

Kaplan-Meier curves showed loss of BAP1, ARID1A and PBRM1 expressions were not significantly associated DFS and OS in patients with ICCs (*P* > 0.05) (Supplementary Table S4) While univariate and multivariate analyses exhibited BAP1 expression loss was correlated to prolonged DFS (*P* = 0.031) and OS (*P* = 0.041) in large duct type of ICCs (Fig. 5). Besides this, none of inactive mutations was statistically associated with DFS or OS in either subtype of ICCs (*P* > 0.05) (Table 4).

### A typical case of ICC patient with IDH1 mutation

**Fig. 6:**
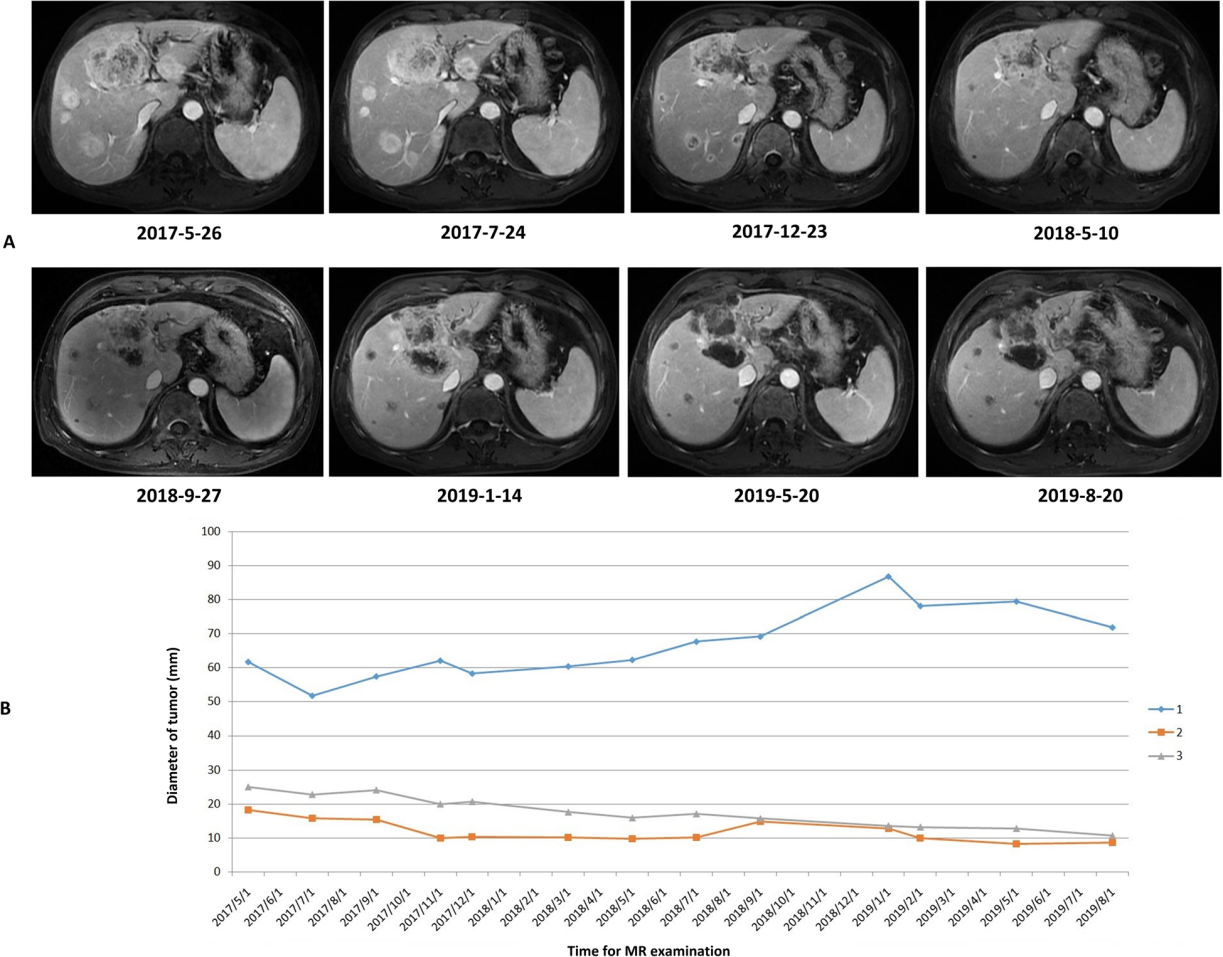
The serial images of abdominal MR (**a**) and diameter curves of 3 representative tumors (**b**) from a typical case during follow-up period. Three representative tumors were marked with corresponding colored arrows in the first MR image

A 61-year-old Chinese man was diagnosed with liver cancer and diffuse intrahepatic and abdominal lymph node metastasis by abdominal magnetic resonance (MR) in May 2017, and subsequently confirmed as ICC (stage IV) with IDH mutation (IDH1-R132C) by biopsy and gene sequencing. He received dasatinib (100 mg, once a day) combined with chemotherapy (gemcitabine, once every three weeks, twenty-five cycles) since June 2017. Abdominal MR in July 2017 showed stable disease (SD). Abdominal MR in January 2019 revealed progressive disease (PD). Therefore, in the same month, this patient was administered with lenvatinib (12 mg, once a day) instead of dasatinib and chemotherapy. Encouragingly, this case still survives with ECOG performance status 1 at the end of this study (September 2019). Figure 6 summarized the patient’s serial images of abdominal MR and diameter curves of representative tumors during follow-up period.

## Discussion

In the present study, we identified 27 (20.8%) and 103 (79.2%) cases as large duct and small duct type ICCs respectively, according to accepted classification criteria [10, 14], but the proportion of large duct type in ICCs was lower than previously reported [10, 15]. There are two possible explanations. Firstly, to ensure the accuracy of this research, we excluded many large duct type ICCs with characteristics similar to those of perihilar carcinoma. Secondly, ICC patients with cholelithiasis, often recognized as large duct type [15], were rarely admitted to our hospital.

Consistent with previous reports [11, 13, 15], we found that ICC patients with large duct type had worse prognosis than those with small duct type in univariate analysis. However, histopathological subtype was not an independent predictor of postoperative survival in multivariate analysis. Furthermore, the small duct type ICCs were closely associated with earlier tumor TNM stage. Accordingly, the bias of tumor stage from both subtypes may account for the inconsistency, and the histological subtype may not be an effective prognostic factor in patients with ICCs.

As reported in previous studies, IDH1/2 mutations were detected in 10.2–28.8% of ICCs [4, 6, 7, 29], and exclusively in small duct type of ICCs [10, 15]. In the present study, we found that 16.1% of patients with ICC harbored IDH1/2 mutation. Meanwhile, the incidence of IDH1/2 mutation in large duct and small duct type of ICCs was 3.7 and 19.4%, respectively. These results are consistent with previous reports. Consequently, the clinical features and prognostic value of IDH1/2 mutations should be investigated in small duct type, rather than in large duct type. However, previous studies have revealed inconsistent results regarding prognostic value of IDH mutation in ICCs. Yuchen Jiao, et al. [4] identified that ICC patients with IDH1/2 mutation had 3-year overall survival of 33% compared to 81% for patients with IDH1/2 wild-type (*P* = 0.0034), but Pu Wang, et al. [7] showed IDH1/2 mutation was significantly associated with improved prognosis in ICCs (4-years tumor recurrence rate was 45.3% vs. 71.5%; *P* = 0.046). At the same time, one study on advanced stage ICCs from Massachusetts General Hospital Cancer Center [29] found that there were no significant differences in DFS and OS between patients with IDH mutation and patients with IDH wild-type. In the present study, univariate and multivariate analyses showed patients with IDH1/2 mutation had significant prolonged DFS and a trend toward increased OS in small duct type of ICCs. Furthermore, IDH1/2 mutations were associated with higher histological differentiation, but not tumor stage. Therefore, these findings suggest that IDH1/2 mutation is a favorable predictor for small duct type ICCs. Due to worse prognosis of large duct type ICCs, cases with IDH1/2 mutation had significantly better DFS and OS than those with IDH1/2 wild-type in total cohort of ICCs. Our results may be account for the conflicting predictive values of IDH1/2 mutation in ICCs described above. In small duct type of ICCs, IDH1/2 mutations were correlated to a trend toward improved OS though the differences were not statistically significant. The inconsistence in DFS and OS maybe ascribed to the development of chemotherapy, immunotherapy, chemotherapy, local regional therapy and combination of different modalities, which obviously prolonged survival even after tumor recurrence. We further analyzed the treatments of ICC patients after recurrence and found that cases with small bile duct type were mostly to be treated with chemotherapy or palliative care. Coincidentally, a total of 9 patients underwent reoperation or radiofrequency ablation (RFA), all of which were classified as IDH wild-type. Therefore, this discrepancy between DFS and OS may be explained by the bias of different treatments after tumor recurrence in two groups. Moreover, the typical case described in methods who reached long-term (20 months) SD after treated with dasatinib also proved that ICC patient with IDH1/2 mutation had a better prognosis, even if the tumor is of advanced stage. In summary, IDH1/2 mutation may be a favorable predictor of small duct type ICCs, and have higher prognostic value for DFS than for OS.

Some investigators have found that IDH1/2 mutation was correlated to several clinical characteristics in ICCs, such as lower level of CA19–9, lower incidence of lymph node metastasis and smaller size of tumors [6, 29]. Interestingly, these clinical features were also observed in the small duct type of ICCs in our study. Since IDH1/2 mutations were typically detected in small duct type of ICCs, we considered these factors (CA19–9, lymph node metastasis and size of tumor) might be associated with small duct type, rather than IDH1/2 mutation. We firstly found that IDH1/2 mutation was strongly associated with decreased preoperative Fe and total bilirubin in small duct type of ICCs. Although the association of IDH1/2 mutation with iron and bilirubin metabolism has not been reported, previous studies have showed that in tricarboxylic acid cycle, iron homeostasis is regulated by haem and citric acid, both of which are closely related to the activity of IDH [30, 31]. Accordingly, the association between IDH1/2 mutation and iron metabolism may provide a new direction for the function study of IDH1/2 mutation in ICCs. Meanwhile, ICCs with IDH1/2 mutation, often categorized as small duct type, localized at the periphery of bile duct, rarely resulting in biliary obstruction in contralateral bile duct, which can explain the relationship between IDH1/2 mutation and lower level of serum total bilirubin.

It should be noted that IDH1/2 mutation is the most frequent mutant type of gliomas and can be detected by either DNA sequencing or IHC methods. Although DNA sequencing is regarded as gold standard, IHC seems to be more accurate, easier to perform and cheaper for detecting IDH1/2 mutation in glioma patients [32]. We firstly used MsMab-1 for detecting IDH1/2 mutation in ICCs by IHC method, and found that the results were basically consistent with those obtained by DNA sequencing. However, IDH1-R132C and IDH1-R132L, two common mutant types in ICCs, cannot be recognized by IHC so far, because their specific antibodies have not been commercially available yet. Therefore, multi-specific antibodies against hotspot mutations of IDH1/2 in ICCs need to be constructed and validated effectively in future.

Surgery remains the mainstream of potentially curative treatment for patients with resectable ICCs. Chemotherapy and radiotherapy are used as first-line therapy for advanced or relapsed ICC patients who are not candidate for surgery. Molecular targeted therapy and immunotherapy are developing very fast in the era of individualized and precision medicine [33]. Because of the low incidence of IDH1/2 mutations (3.7%), postoperative chemotherapy is recommended for ICC patients with large duct type without the need for gene sequencing. In contrast, due to the high incidence of IDH1/2 mutations (19.4%) in small bile duct type of ICCs, DNA sequencing should be recommend to detect IDH1/2 mutation and choose appropriate adjuvant therapies. If IDH1/2 mutation is detected, s small molecular medications that target IDH1/2 (dasatinib, ivosidenib or enasidenib) could be a promising treatment strategy [34–36]. With the development of specific antibodies against mutant IDH1/2, IHC will be an easy and cheap way to detect IDH1/2 mutation.

In addition, the clinical implications of other most frequent mutations (including BAP1, ARID1A and PBRM1) were investigated in each subtype of ICCs. Surprisingly, these inactive mutations were not significantly associated DFS and OS in small duct type of ICCs. Nonetheless, only BAP1 expression loss was correlated to prolonged DFS and OS in the large duct type of ICCs. Many studies [4, 25, 28] showed that these factors have no prognostic value in ICCs, but Sarcognato S et al. [8] found that the retained expression of BAP1 was correlated to poor prognosis, while another two studies [8, 9] got to conflicting conclusions about predictive value of ARID1A in ICCs. Accordingly, our study may explain the inconsistency in the prognostic value of BAP1: loss of BAP1 expression was correlated to improved survival in large duct type ICCs rather than in small duct type ICCs. When two subtypes were combined into one group, BAP1 can be considered as a favorable or irrelevant predictor in ICCs according to the ratio of large duct to small duct type ICCs.

There were limitations in this study. Firstly, the sample size is too small, especially for patients with large duct type ICC. This is why KRAS mutation, frequent in large duct type ICC, was not included in this study. Secondly, FGFR2-fusion, another frequent mutation in ICCs, was not investigated in this research.

## Conclusion

IDH1/2 mutation is a favorable predictor and may be related to iron metabolism in small duct type of ICCs. Furthermore, we suggest that the detection of IDH1/2 mutation is indispensable to determine targeted therapy in small duct type ICCs, while it is not necessary in large duct of ICCs. MsMab-1 is a relatively effective multi-specific antibody against IDH1/2 mutant in ICCs. BAP1 expression loss was correlated with improved prognosis only in large duct type ICCs.

## Supplementary information

**Additional file 1.**

## Data Availability

The datasets used and/or analysed during the current study are available from the corresponding author on reasonable request.

## Abbreviations

ICC: Intrahepatic cholangiocarcinoma
OS: Overall survival
IDH: Isocitrate dehydrogenase
α-KG: α-ketoglutarate
2HG: 2-hydroxyglutarate
IHC: Immunohistochemistry
FFPE: Formalin-fixed paraffin-embedded
HE: Hematoxylin-eosin staining
TMA: Tissue microarray
DFS: Disease-free survival
HR: Hazard ratios
CIs: Confidence intervals
TBIL: Total bilirubin
Fe: Ferritin
ALT: Alanine aminotransferase
AST: Aspartate aminotransferase
IBIL: Indirect bilirubin
MR: Magnetic resonance
SD: Stable disease
RFA: Radiofrequency ablation
PD: Progressive disease
DBIL: Direct bilirubin
CEA: Carcinoembryonic antigen
