# Supplementary material for: Distinct clinical and prognostic implication of IDH1/2 mutation and other most frequent mutations in large duct and small duct subtypes of intrahepatic cholangiocarcinoma
Source: BMC Cancer. 2020 Apr 15;20:318. doi: 10.1186/s12885-020-06804-6 (PMC7161164; doi:10.1186/s12885-020-06804-6)
Supplement: Supplementary file 1 — Additional file 1. [file 12885_2020_6804_MOESM1_ESM.docx]

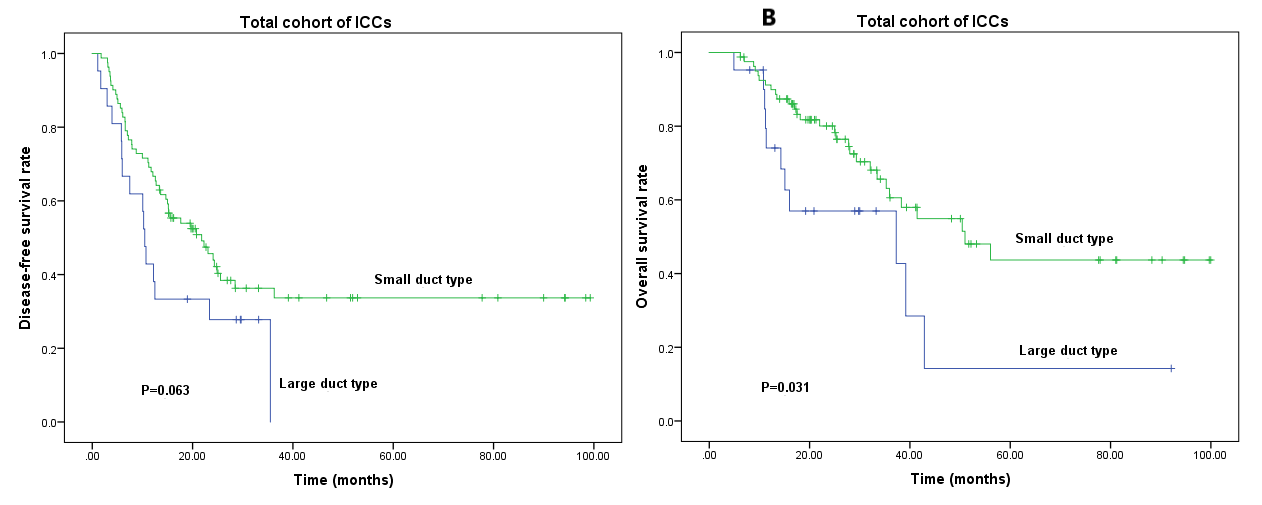


**Figure S1.** Kaplan-Meier curves showing patients with small duct type had improved DFS (A) and OS (B) in total cohorts of ICCs.

**Table S1.** Results of the ICC subclassification in the first round.

| **Subtype** | **HE** | | |  | **AB** | | |  | **S100P** | | | | |
| --- | --- | --- | --- | --- | --- | --- | --- | --- | --- | --- | --- | --- | --- |
|  | Large | Small | underdetermined |  | 0 | 1 | 2 |  | 0 | 1 | 2 | 3 | 4 |
| **Large duct** | 12 | 0 | 0 |  | 0 | 1 | 11 |  | 0 | 0 | 0 | 2 | 10 |
| **Small duct** | 0 | 73 | 0 |  | 72 | 1 | 0 |  | 58 | 15 | 0 | 0 | 0 |
| **intermediate** | 0 | 13 | 32 |  | 23 | 12 | 10 |  | 10 | 7 | 6 | 17 | 5 |

**Table S2.** Results of the intermediate type ICC subclassification in the second round.

| **Subtype** | **HE** | | |  | **AB** | | |  | **S100P** | | | | |
| --- | --- | --- | --- | --- | --- | --- | --- | --- | --- | --- | --- | --- | --- |
|  | Large | Small | underdetermined |  | 0 | 1 | 2 |  | 0 | 1 | 2 | 3 | 4 |
| **Large duct** | 0 | 0 | 15 |  | 3 | 5 | 7 |  | 0 | 2 | 0 | 8 | 5 |
| **Small duct** | 0 | 13 | 17 |  | 20 | 7 | 3 |  | 10 | 5 | 6 | 9 | 0 |

**Table S3.** Comparisons of clinicopathological characteristics between large duct and small duct type of ICCs.

| **Clinical variables** | **Large duct type** | **Small duct type** | **P-value** |
| --- | --- | --- | --- |
| **Age** (years) | |  | 0.071 |
| <65 | 17 (63.0%) | 82 (79.6%) |  |
| ≥65 | 10 (37.0%) | 21 (20.4%) |  |
| **Gender** |  |  | 0.233 |
| Male | 12 (44.4%) | 59 (57.3%) |  |
| Female | 15 (55.6%) | 44 (42.7%) |  |
| **CA19-9** (U/ml) | |  | **0.002** |
| <39 | 6 (22.2%) | 57 (55.9%) |  |
| ≥39 | 21 (77.8%) | 45 (44.1%) |  |
| **Lymphadenectomy** | |  | **0.002** |
| Negative | 11 (40.7%) | 75 (72.8%) |  |
| Positive | 16 (59.3%) | 28 (27.2%) |  |
| **Nerve invasion** | |  | **0.025** |
| Negative | 16 (61.5%) | 82 (82.0%) |  |
| Positive | 10 (38.5%) | 18 (18.0%) |  |
| **Satellite lesions** | |  | **0.009** |
| Negative | 18 (66.7%) | 92 (89.3%) |  |
| Positive | 9 (33.3%) | 11 (10.7%) |  |
| **Diameter of tumor** (cm) | |  | **0.021** |
| ＜5 | 18(66.7%) | 43(41.7%) |  |
| ≥5 | 9(33.3%) | 60(58.3%) |  |
| **pT classification** | |  | **0.041** |
| pT1 | 7 (25.9%) | 45 (43.7%) |  |
| pT2 | 13 (48.1%) | 47 (45.6%) |  |
| pT3 | 3 (11.1%) | 2 (1.9%) |  |
| pT4 | 4 (14.8%) | 9 (8.7%) |  |
| **pN classification** | |  | 0.117 |
| N0 | 11 (50.0%) | 34 (69.4%) |  |
| N1 | 11 (50.0%) | 15 (30.6%) |  |
| **pM classification** | |  | **0.019** |
| M0 | 19 (70.4%) | 93 (90.3%) |  |
| M1 | 8 (29.6%) | 10 (9.7%) |  |
| **TNM stage** |  |  | **0.004** |
| I | 1 (4.5%) | 14 (26.9%) |  |
| II | 6 (27.3%) | 19 (36.5%) |  |
| III | 8 (36.4%) | 10 (19.2%) |  |
| IV | 7 (31.8%) | 9 (17.3%) |  |

**Table S4.** Univariate and multivariate analyses for prognostic value of frequent mutations in ICCs

| **clinical variables** | **DFS** | |  | **OS** | |
| --- | --- | --- | --- | --- | --- |
|  | Univariate  P-value | Multivariate P-value/  HR (95% CI) |  | Univariate  P-value | Multivariate P-value/  HR (95% CI) |
| **IDH1/2** | **0.006** | **0.026** |  | **0.031** | **0.038** |
| Mutant vs. wild |  | 2.915(1.136-7.477) |  |  | 3.593(1.074-12.018) |
| **BAP1** | 0.475 |  |  | 0.956 |  |
| Loss vs. retained |  |  |  |  |  |
| **ARID1A** | 0.355 |  |  | 0.629 |  |
| Loss vs. retained |  |  |  |  |  |
| **PBRM1** | 0.426 |  |  | 0.449 |  |
| Loss vs. retained |  |  |  |  |  |

**Table S5.** Loss of BAP1, ARID1A and PBRM1 expressions in ICCs

| **Factors** | **Score 0** | **Score 1** | **Score 2** |
| --- | --- | --- | --- |
| **BAP1** | 47(36.2%) | 11(8.5%) | 72(55.4%) |
| **ARID1A** | 24(18.5%) | 4(3.1%) | 102(78.5%) |
| **PBRM1** | 42(32.3%) | 3(2.3%) | 85(65.4%) |

**Table S6.** The correlation among BAP1, ARID1A and PBRM1 expression loss

| **Mutant** | | **ARID1A** | | |  | **PBRM1** | | |
| --- | --- | --- | --- | --- | --- | --- | --- | --- |
| **factors** | | loss | retained | **P-value** |  | loss | retained | **P-value** |
| **BAP1** | loss | 18 | 40 | **0.018** |  | 34 | 24 | **0.000** |
|  | retained | 10 | 62 |  |  | 11 | 61 |  |
| **PBRM1** | loss | 15 | 30 | **0.017** |  |  |  |  |
|  | retained | 13 | 72 |  |  |  |  |  |

**Table S7.** Comparisons of clinicopathologic characteristics between BAP1 expression loss and retained in ICCs and their subtypes

| **Clinical variables** | **Total cohort** | | |  | **Large bile duct** | | |  | **Small bile duct** | | |
| --- | --- | --- | --- | --- | --- | --- | --- | --- | --- | --- | --- |
|  | BAP1 loss | BAP1 retained | **P-valve** |  | BAP1 loss | BAP1 retained | **P-valve** |  | BAP1 loss | BAP1 retained | **P-valve** |
| **ALT** (U/L) | |  | 0.908 |  |  |  | **0.047** |  |  |  | 0.211 |
| <40 | 43(74.1%) | 52(73.2%) |  |  | 10(83.3%) | 6(40.0%) |  |  | 33(71.7%) | 46(82.1%) |  |
| ≥40 | 15(25.9%) | 19(26.8%) |  |  | 2(16.7%) | 9(60.0%) |  |  | 13(28.3%) | 10(17.9%) |  |
| **AST** (U/L) | |  | 0.495 |  |  |  | **0.014** |  |  |  | 0.326 |
| <40 | 47(81.0%) | 54(76.1%) |  |  | 11(91.7%) | 6(40.0%) |  |  | 36(78.3%) | 48(85.7%) |  |
| ≥40 | 11(19.0%) | 17(23.9%) |  |  | 1(8.3%) | 9(50.0%) |  |  | 10(21.7%) | 8(14.3%) |  |
| **TBIL** (umol/L) | |  | 0.861 |  |  |  | **0.001** |  |  |  | 0.117 |
| <21 | 36(62.1%) | 43(60.6%) |  |  | 10(83.3%) | 3(20.0%) |  |  | 26(56.5%) | 40(71.4%) |  |
| ≥21 | 22(37.9%) | 28(39.4%) |  |  | 2(16.7%) | 12(80.0%) |  |  | 20(43.5%) | 16(28.6%) |  |
| **Diameter of tumor** | |  | 0.136 |  |  |  | 0.217 |  |  |  | **0.013** |
| ＜5 | 23(39.7%) | 38(52.8%) |  |  | 10(83.3%) | 8(53.3%) |  |  | 13(28.3%) | 30(52.6%) |  |
| ≥5 | 35(60.3%) | 34(47.2%) |  |  | 2(16.7%) | 7(46.7%) |  |  | 33(71.7%) | 27(47.4%) |  |
| **Histological differentiation** | |  | 0.356 |  |  |  | **0.014** |  |  |  | 0.793 |
| Low | 23(41.4%) | 35(49.3%) |  |  | 1(8.3%) | 8(57.1%) |  |  | 22(50.0%) | 27(47.4%) |  |
| Moderately to high | 33(58.9%) | 36(50.7%) |  |  | 11(91.7%) | 6(42.9%) |  |  | 22(50.0%) | 30(52.6%) |  |
| **Histological subtype** | |  | 0.984 |  |  |  |  |  |  |  |  |
| Large duct | 12(20.7%) | 15(20.8%) |  |  |  |  |  |  |  |  |  |
| Small duct | 46(79.3%) | 57(79.2%) |  |  |  |  |  |  |  |  |  |

**Table S8.** Comparisons of clinicopathologic characteristics between ARID1A expression loss and retained in ICCs and their subtypes

| **Clinical variables** | **Total cohort** | | |  | **Large bile duct** | | |  | **Small bile duct** | | |
| --- | --- | --- | --- | --- | --- | --- | --- | --- | --- | --- | --- |
|  | ARID1A loss | ARID1A retained | **P-valve** |  | ARID1A loss | ARID1A retained | **P-valve** |  | ARID1A loss | ARID1A retained | **P-valve** |
| **ALT**  (U/L) | |  | **0.034** |  |  |  | 0.692 |  |  |  | **0.006** |
| ＜40 | 25(89.3%) | 70(69.3%) |  |  | 6(66.7%) | 10(55.6%) |  |  | 19(100.0%) | 60(72.3%) |  |
| ≥40 | 3(10.7%) | 31(30.7%) |  |  | 3(33.3%) | 8(44.4%) |  |  | 0(0.0%) | 23(27.7%) |  |
| **AST** (U/L) | |  | **0.035** |  |  |  | 0.406 |  |  |  | **0.021** |
| ＜40 | 26(92.9%) | 75(74.3%) |  |  | 7(77.8%) | 10(55.6%) |  |  | 19(100.0%) | 65(78.3%) |  |
| ≥40 | 2(7.1%) | 26(25.7%) |  |  | 2(22.2%) | 8(44.4%) |  |  | 0(0.0%) | 18(21.7%) |  |
| **Liver cirrhosis** | |  | **0.005** |  |  |  | 0.636 |  |  |  | **0.008** |
| Negative | 25(89.3%) | 62(60.8%) |  |  | 8(88.9%) | 14(77.8%) |  |  | 17(89.5%) | 48(57.1%) |  |
| Positive | 3(10.7%) | 40((39.2%) |  |  | 1(11.1%) | 4(22.2%) |  |  | 2(10.5%) | 36(42.9%) |  |
| **Diameter of tumor** (cm) | |  | 0.180 |  |  |  | 1.000 |  |  |  | **0.043** |
| ＜5.0 | 10(35.7%) | 51(50.0%) |  |  | 6(66.7%) | 12(66.7%) |  |  | 4(21.1%) | 39(46.4%) |  |
| ≥5.0 | 18(64.3%) | 51(50.0%) |  |  | 3(33.3%) | 6(33.3%) |  |  | 15(78.9%) | 45(53.6%) |  |
| **N classification** | |  | **0.009** |  |  |  | 0.183 |  |  |  | **0.021** |
| N0 | 16(88.9%) | 29(54.7%) |  |  | 6(75.0%) | 5(35.7%) |  |  | 10(100.0%) | 24(61.5%) |  |
| N1 | 2(11.1%) | 24(45.3%) |  |  | 2(25.0%) | 9(64.3%) |  |  | 0(0.0%) | 15(38.5%) |  |
| **TNM stage** | |  | **0.046** |  |  |  | 0.343 |  |  |  | **0.040** |
| I～II | 14(73.7%) | 26(47.3%) |  |  | 4(50.0%) | 3(21.4%) |  |  | 10(90.9%) | 23(56.1%) |  |
| III～IV | 5(26.3%) | 29(52.7%) |  |  | 4(50.0%) | 11(78.6%) |  |  | 1(9.1%) | 18(43.9%) |  |
| **Histological subtype** | |  | 0.094 |  |  |  |  |  |  |  |  |
| Large duct | 9(32.1%) | 18(17.6%) |  |  |  |  |  |  |  |  |  |
| Small duct | 19(67.9%) | 84(82.4%) |  |  |  |  |  |  |  |  |  |

**Table S9.** Comparisons of clinicopathologic characteristics between PBRM1 expression loss and retained in ICCs and their subtypes

| **Clinical variables** | **Total cohort** | | |  | **Large bile duct** | | |  | **Small bile duct** | | |
| --- | --- | --- | --- | --- | --- | --- | --- | --- | --- | --- | --- |
|  | PBRM1 loss | PBRM1 retained | **P-valve** |  | PBRM1 loss | PBRM1 retained | **P-valve** |  | PBRM1 loss | PBRM1 retained | P-valve |
| **AST** (U/L) | | | 0.091 |  |  |  | **0.018** |  |  |  | 0.582 |
| ＜40 | 39(86.7%) | 62(73.8%) |  |  | 10(90.9%) | 7(43.8%) |  |  | 29(85.3%) | 55(80.9%) |  |
| ≥40 | 6(13.3%) | 22(26.2%) |  |  | 1(9.1%) | 9(56.3%) |  |  | 5(14.7%) | 13(19.1%) |  |
| **TBIL**  (umol/L) | | | 0.109 |  |  |  | **0.022** |  |  |  | 0.707 |
| ＜21 | 40(88.9%) | 65(77.4%) |  |  | 11(100.0%) | 9(56.3%) |  |  | 29(85.3%) | 56(82.4%) |  |
| ≥21 | 5(11.1%) | 19(22.6%) |  |  | 0(0.0%) | 7(43.8%) |  |  | 5(14.7%) | 12(17.6%) |  |
| **DBIL**  (umol/L) | | | 0.832 |  |  |  | **0.034** |  |  |  | 0.187 |
| ＜3.4 | 27(60.0%) | 52(61.9%) |  |  | 8(72.7%) | 5(31.3%) |  |  | 19(55.9%) | 47(69.1%) |  |
| ≥3.4 | 18(40.0%) | 32(38.1%) |  |  | 3(27.3%) | 11(68.8%) |  |  | 15(44.1%) | 21(30.9%) |  |
| **CA19-9** (U/ml) | | | 0.465 |  |  |  | **0.027** |  |  |  | 0.091 |
| ＜39 | 20(44.4%) | 43(51.2%) |  |  | 5(45.5%) | 1(6.3%) |  |  | 15(44.1%) | 42(61.8%) |  |
| ≥39 | 25(55.6%) | 41(48.8%) |  |  | 6(54.5%) | 15(93.8%) |  |  | 19(55.9%) | 26(38.2%) |  |
| **Liver cirrhosis** | | | 0.056 |  |  |  | 1.000 |  |  |  | **0.048** |
| Negative | 35(77.8%) | 52(61.2%) |  |  | 9(81.8%) | 13(81.3%) |  |  | 26(76.5%) | 39(56.5%) |  |
| Positive | 10(22.2%) | 33(38.8%) |  |  | 2(18.2%) | 3(18.8%) |  |  | 8(23.5%) | 30(43.5%) |  |
| **Histological subtype** | | | 0.452 |  |  |  |  |  |  |  |  |
| Large duct | 11(24.4%) | 16(18.8%) |  |  |  |  |  |  |  |  |  |
| Small duct | 34(75.6%) | 69(81.2%) |  |  |  |  |  |  |  |  |  |
